# A Water-Stable 2-Fold Interpenetrating cds Net as a Bifunctional Fluorescence-Responsive Sensor for Selective Detection of Cr(III) and Cr(VI) Ions

**DOI:** 10.3390/nano12010158

**Published:** 2022-01-03

**Authors:** Meng-Jung Tsai, Kuo-Shun Liao, Jing-Yun Wu

**Affiliations:** Department of Applied Chemistry, National Chi Nan University, Nantou 545, Taiwan; s97324905@mail1.ncnu.edu.tw (M.-J.T.); aasd6307@gmail.com (K.-S.L.)

**Keywords:** chromium, coordination polymer, fluorescence sensor, interpenetrating

## Abstract

Reactions of ZnSO_4_∙7H_2_O, *N*-(pyridin-3-ylmethyl)-4-(pyridin-4-yl)-1,8-naphthalimide (NI-mbpy-34), and 5-bromobenzene-1,3-dicarboxylic acid (Br-1,3-H_2_bdc) afforded a luminescent coordination polymer, [Zn(Br-1,3-bdc)(NI-mbpy-34)]*_n_* (**1**), under hydro(solvo)thermal conditions. Single-crystal X-ray structure analysis revealed that **1** features a three-dimensional (3-D) 2-fold interpenetrating **cds** (or CdSO_4_) net topology with the point symbol of (6^5^·8), where the Zn(II) centers are considered as 4-connected square-planar nodes. X-ray powder diffraction (XRPD) patterns and thermogravimetric (TG) analysis confirmed that **1** shows high chemical and thermal stabilities. Notably, **1** displayed solvent dependent photoluminescence properties; the fluorescence intensity and emission maximum of **1** in different solvent suspensions varied when a solvent was changed. Furthermore, the H_2_O suspension of **1** exhibited blue fluorescence emission and thus can be treated as a selective and sensitive fluorescent probe for turn-on detection of Cr^3+^ cations through absorbance caused enhancement (ACE) mechanism and turn-off detection of Cr_2_O_7_^2−^/CrO_4_^2−^ anions through collaboration of the absorption competition and energy transfer process, with limit of detection (LOD) as low as μM scale.

## 1. Introduction

The monitoring and detection of chemical pollutants and/or controlled chemicals in complicated samples are very important tasks in managing the environment, water resources, and the food industry. Among various conventional instrumental techniques, fluorescence sensing responding to fluorescence turn on, turn off, or ratiometric signal, has attracted immense attention in recent years because of its particular aspects such as economics, user-friendliness, short response time, visualization, monitoring in real-time, excellent sensitivity, and high selectivity [1,2,3,4]. Various advanced fluorophore materials, including organic dyes [5,6], porous organic polymers [7], quantum dots (QDs) [8,9], carbon dots (CDs) [1,2], nanoparticles (NPs) [3,10], lanthanide organic/inorganic hybrid materials (LHMs) [11], and metal–organic frameworks/coordination polymers (MOFs/CPs) [12,13,14] have emerged.

Chromium existing as Cr(III) and Cr(VI) oxidation states in the aquatic environments can directly contaminate the soil and aquatic systems. As an essential trace biological element in humans, Cr(III) is considered to be harmless and safe. However, excessive Cr(III) may combine with DNA to cause mutations and malignant cells [10,15,16,17]. Cr(VI) shows high carcinogenicity and mutagenicity and can cause allergic reactions, hereditary genetic defects and various types of cancers that adversely affect human health [17,18,19]. The World Health Organization (WHO) has claimed a permissible limit of 50 μg/L for Cr(VI) in drinking water [20]. Lately, MOF/CP-based, fluorescence-sensory materials have been actively pursued as excellent platforms for the flourishing utilization in detection of Cr(III) and Cr(VI) ions though fluorescence quenching (turn off) effect [15,21,22,23,24,25,26,27,28,29,30,31,32,33,34,35,36,37,38,39,40,41,42,43,44,45]. However, there are still rare examples to achieve the detection of Cr(III) via the fluorescence enhancement (turn on) response [15,43,44,45,46,47,48] and fluorescence shift (ratiometric) effect [41,42,43]. 

As part of our ongoing work in fluorescence detection of hazardous chemical contaminants [39,40,41,42,43,44,49,50,51,52], we acquired, herein, a Zn(II)-based luminescent coordination polymer, namely [Zn(Br-1,3-bdc)(NI-mbpy-34)]*_n_* (**1**, Br-1,3-bdc = 5-bromobenzene-1,3-dicarboxylate; NI-mbpy-34 = *N*-(pyridin-3-ylmethyl)-4-(pyridin-4-yl)-1,8-naphthalimide), featuring a three-dimensional (3-D) 2-fold interpenetrating **cds** net. Of note, coordination polymer **1** exhibited fluorescence emissions in solid-state and solvent suspensions, being a bifunctional fluorescence sensor for sensitively and selectively detecting chromium(III) cations and chromium(VI) oxyanions. 

## 2. Experimental Section

### 2.1. Materials and Methods

All of the chemicals and solvents were acquired from market sources and used without further processing. Ligand NI-mbpy-34 was synthesized according to the previously reported literature [44]. The thermal analysis was conducted by a Thermo Cahn VersaTherm HS TG analyzer (Thermo, Newington, NH, USA) from 25 to 900 °C at a heating rate of 5 °C/min under a flow of nitrogen. The X-ray powder diffraction (XRPD) patterns were measured in the 2*θ* range of 5–50° by a Shimadzu XRD-7000 diffractometer (Shimadzu, Kyoto, Japan) using Cu Kα radiation (*λ* = 1.5406 Å) operating at 30 kV and 30 mA. Infrared (IR) spectroscopy was tested in a Perkin-Elmer Frontier Fourier transform infrared spectrometer (Perkin-Elmer, Taipei, Taiwan), and the region 4000–500 cm^−1^ was recorded with attenuated total reflection (ATR) technique. UV-Vis absorption spectra were obtained on a JASCO V-750 UV/VIS spectrophotometer (JASCO, Tokyo, Japan) at room temperature. The solid-state and solution fluorescence spectra were measured on a Hitachi F7000 fluorescence spectrophotometer (Hitachi, Tokyo, Japan) at room temperature, with the excitation and emission slits of 5 nm × 5 nm and a scan rate of 1200 nm/min. A 150 W xenon arc lamp was used as an exciting light source. Elemental analyses of C, H, and N were performed on a Vario EL III elemental analyzer (Elementar, Langenselbold, Germany). X-ray photoelectron spectroscopy (XPS) was measured by an ULVAC-PHI PHI 5000 VersaProbe/Scanning ESCA Microprobe instrument (ULVACPHI Inc., Kanagawa, Japan). 

### 2.2. Synthesis of [Zn(Br-1,3-bdc)(NI-mbpy-34)]_n_ (***1***)

NI-mbpy-34 (9.1 mg, 0.025 mmol) was dissolved in 2 mL of *N*,*N*′-dimethylformamide (DMF); ZnSO_4_∙7H_2_O (14.3 mg, 0.050 mmol) was dissolved in 2 mL of H_2_O; Br-1,3-H_2_bdc (12.3 mg, 0.050 mmol) was dissolved in 1 mL of DMF. The above-mentioned solutions were sequentially added to a 23 mL Teflon-lined stainless steel reactor placed in an autoclave. This was sealed and then heated to 80 °C for 6 h and kept at 80 °C for 48 h. After slowly cooling to 30 °C for 36 h, the mixture was washed with distilled water and ethanol, and yellowish crystals were filtered off and dried. The yield based on NI-mbpy-34 was about 60%. IR (ATR, cm^−1^): 3071, 1617, 1322, 1462, 990, 884, 723. Anal. Calcd for C_31_H_18_BrN_3_O_6_Zn: C, 55.21; H, 2.67; N, 6.23%. Found: C, 54.90; H, 2.65; N, 6.20%.

### 2.3. Single-Crystal X-ray Structure Determinations

The single-crystal data taken at 150(2) K for **1** were collected on a Bruker D8 Venture diffractometer with a graphite monochromated Mo Kα radiation (*λ* = 0.71073 Å) and a PHOTO100 CMOS detector. The structures were solved by direct methods using SHELXTL [53] and refined on *F*^2^ by the full-matrix least-squares using the SHELXL-2014/7 [54] and WINGX [55]. Non-hydrogen atoms were confirmed by successive difference Fourier syntheses and were refined with anisotropic displacement parameters. The hydrogen atoms were produced theoretically on their calculated positions and refined with isotropic displacement parameters set to 1.2*U_eq_* of the attached atom. The single-crystal data and refinement parameters of **1** are summarized in Table 1. CCDC 1991626 (**1**) contains the supplementary crystallographic data for this paper. These data can be obtained free of charge from the Cambridge Crystallographic Data Centre via www.ccdc.cam.ac.uk/data_request/cif (23 December 2022).

### 2.4. Fluorescence Measurements

Finely ground powders of **1** (1 mg) were suspended in various solvents (3 mL) including dichloromethane (CH_2_Cl_2_), *N*,*N*′-dimethylacetamide (DMAc), *N*,*N*′-dimethylformamide (DMF), H_2_O, methanol (CH_3_OH), and toluene. The prepared suspensions were ultrasonicated via pulsed ultrasound for 10 min and then agitated for further 30 min to yield more stable suspensions. 

The H_2_O suspensions of **1** were utilized to conduct fluorescence sensing experiments. Aqueous solutions of metal ions, including AgNO_3_, Al(NO_3_)_3_, Mg(NO_3_)_2_, Ca(NO_3_)_2_, Co(NO_3_)_2_, Cr(NO_3_)_3_, Cu(NO_3_)_2_, Fe(NO_3_)_3_, NaNO_3_, KNO_3_, Mn(NO_3_)_2_, Ni(NO_3_)_2_, and Pb(NO_3_)_2_, and anions, including NaF, KCl, KBr, KI, KClO_4_, K_2_CO_3_, K_2_Cr_2_O_7_, K_2_CrO_4_, KNO_3_, and K_3_PO_4_ were prepared with concentration of 0.10 M for fluorescence sensing studies. 

Qualitative studies were carried out by adding 0.10 M analyte (30 μL) into the well-prepared H_2_O suspensions of **1**; then, the fluorescence spectra were recorded after waiting for 3 min. Anti-interference studies were conducted on a series of competition experiments with addition of the solution of different perturbed analytes (0.10 M, 30 μL) followed by the targeted analyte (0.10 M, 30 μL) into the H_2_O suspensions. In each step, the fluorescence spectra were recorded.

The fluorescence quantitative titration experiments were performed with the gradual addition of analytes in aqueous solutions (0.10 M), and then the fluorescence spectra were monitored. The Stern–Volmer equation: *I*_0_/*I* = 1 + *K*_sv_[Q], where *I*_0_ and *I* denote the fluorescence intensities before and after the addition of analytes, respectively, *K*_sv_ is the Stern–Volmer quenching constant (M^−1^), and [Q] is the concentration of analyte (mM), was applied to quantitatively analyze the fluorescence quenching effect. 

Limit of detection (LOD) determinations were performed at low concentrations of analyte. Prior to the fluorescence titration, five blank measurements of fluorescence for the H_2_O suspensions of **1** were carried out for determining the standard deviation (*σ*). LODs were calculated using the equation: LOD = 3*σ*/*k*, where *k* represents the absolute value of the slope of the calibration curve. 

## 3. Results and Discussion

### 3.1. Crystal Structure of [Zn(Br-1,3-bdc)(NI-mbpy-34)]_n_ (***1***)

**Figure 1:**
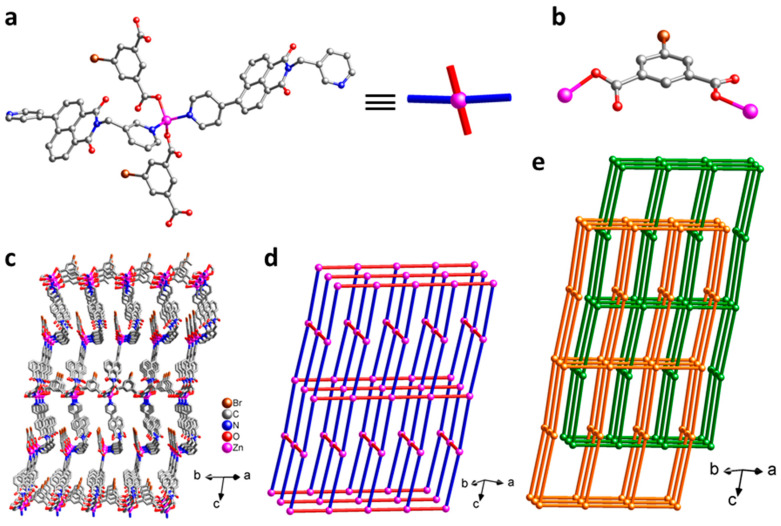
Crystal structure of **1**: (**a**) the coordination environment around the Zn(II) center and schematic representation of the 4-connected node; (**b**) the coordination mode of Br-1,3-bdc^2−^ dianion; (**c**) a single 3-D framework; (**d**) schematic representation of the 4-connected **cds** network with the point symbol of (6^5^·8); (**e**) 2-fold interpenetrating **cds** networks.

Single-crystal X-ray structure analysis reveals that the crystal structure of **1** belongs to the monoclinic space group *C*2/*c*. There is one cationic Zn(II) center, one fully-deprotonated Br-1,3-bdc^2−^ anion, and one neutral NI-mbpy-34 ligand in the asymmetric unit. The Zn(II) center is surrounded by two oxygen atoms of two carboxylate groups from two distinct Br-1,3-bdc^2−^ ligands and two nitrogen atoms of one 3-pyridyl (imide end) and one 4-pyridyl (naphthalene end) groups from two distinct NI-mbpy-34 ligands to adopt a {ZnO_2_N_2_} tetrahedral geometry (Figure 1a). The anionic Br-1,3-bdc^2−^ ligand has a μ_2_-Br-1,3-bdc-κO:κO mode to bridge two Zn(II) centers; each of the two carboxylate groups is in a monodentate-κO coordination mode (Figure 1b). The Zn(II) centers are connected by the anionic Br-1,3-bdc^2−^ and the neutral NI-mbpy-34 ligands to form a three-dimensional (3-D) porous framework (Figure 1c). If the Zn(II) centers are considered as 4-connected square-planar nodes and both the Br-1,3-bdc^2−^ and NI-mbpy-34 ligands are considered as linear linkers (Figure 1a), the 3-D framework of **1** can be simplified as a 4-connected **cds** (or CdSO_4_) net topology with the point symbol of (6^5^·8) (Figure 1d). The potential voids of the single **cds** network are occupied by the other independent identical framework via interpenetration in opposite orientation to generate a 2-fold interpenetrating net (Figure 1e), leaving insufficient solvent accessible voids. Notably, two neighboring naphthalimide skeletons in the two independent equivalent **cds** frameworks are nearly parallel in a head-to-tail manner and the distance between them is about 3.50 Å (Figure S1), suggesting significant π–π interactions.

### 3.2. X-ray Powder Diffraction (XRPD) Patterns and Chemical Stability

**Figure 2:**
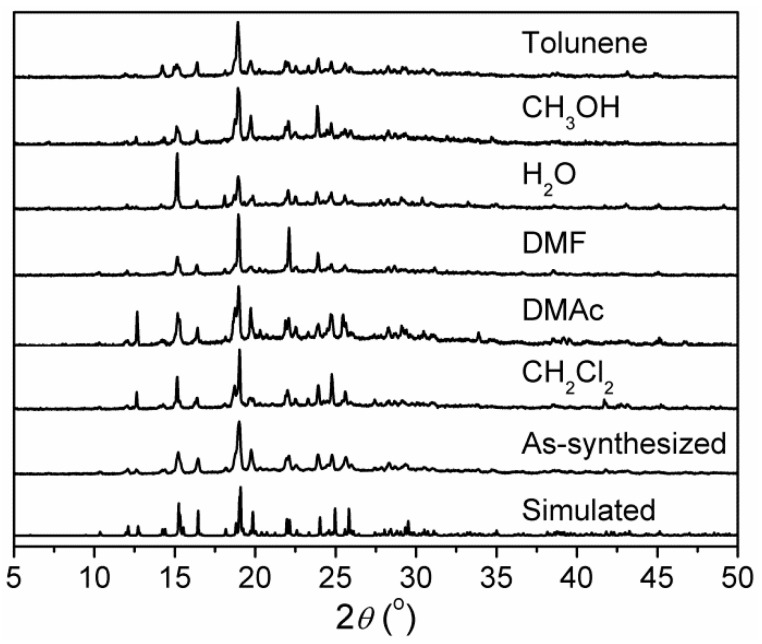
Simulated XRPD pattern of **1** and XRPD patterns of as-synthesized **1** and **1** immersed in different solvents for 24 h.

X-ray powder diffraction (XRPD) patterns of as-synthesized **1** are in agreement with the simulated patterns calculated from single-crystal X-ray diffraction data (Figure 2), confirming the phase purity of bulky samples. Further, the chemical stability of **1** in different solvents was checked. After immersing in dichloromethane (CH_2_Cl_2_), *N*,*N*′-dimethylacetamide (DMAc), *N*,*N*′-dimethylformamide (DMF), H_2_O, methanol (CH_3_OH), and toluene for 24 h, the XRPD patterns of the solvent-treated samples showed that the characteristic peaks match well with those of the XRPD pattern of as-synthesized **1** and that simulated from the single crystal data, although the peak intensities are somewhat different (Figure 2). This demonstrates that the original framework of **1** can retain a high crystallinity after immersion in solvents, confirming its high stability. 

### 3.3. Thermal Properties

The thermal properties of **1** were evaluated from the thermogravimetric (TG) analysis. As a representative, the TG analysis plot of **1** shows no weight loss before 378 °C (Figure S2), indicating high thermal stability. Then a two-step decomposition of the framework occurred, which was ended upon heating to ca. 640 °C. During the decomposition, bromide might react with divalent zinc to generate ZnBr_2_ (b.p. = 697 °C), which escaped at higher temperature. The remaining residue of 6.2% was reasonably assigned to the ZnO component (calcd 6.0%). 

### 3.4. Photoluminescence Properties

Previous research has shown that NI-mbpy-34 is highly emissive and can be a luminescence source for coordination polymers due to its highly conjugated π-electron system [44]. In solid-state, NI-mbpy-34 showed emission band(s) in the region of 400–600 nm with maximum at 462 nm upon excitation at *λ*_ex_ = 370 nm, while Br-1,3-H_2_bdc displayed only an extremely weak emission band upon excitation at *λ*_ex_ = 360 nm (Figure S3). When excited at *λ*_ex_ = 306 nm, **1** exhibited solid-state fluorescence with two emission peaks centered at 444 nm and 504 nm. From the band position and shape, the emissions were tentatively attributed to the ligand-centered emission of NI-mbpy-34 perturbed by metal coordination. 

**Figure 3:**
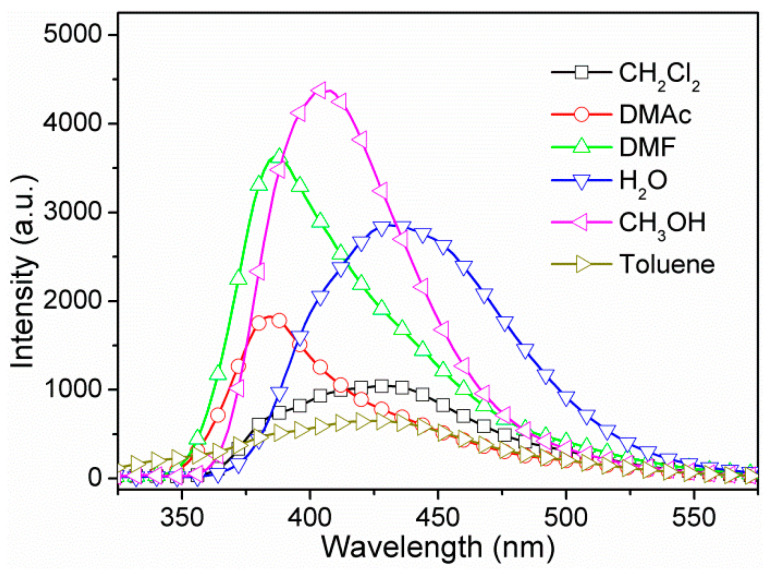
Fluorescence emission spectra of **1** in suspension-phase of different solvents.

Subsequently, the fluorescence properties of **1** in different solvent suspensions, such as CH_2_Cl_2_, DMAc, DMF, H_2_O, CH_3_OH, and toluene were also investigated (Figure 3). We observed that the fluorescence intensity and emission maximum of **1** in different solvent suspensions varied as the solvent was changed, implying solvent-dependent photoluminescence properties. Upon excitation, **1** emitted strong fluorescence emissions in CH_3_OH and DMF suspensions, moderate emissions in H_2_O and DMAc suspensions, and weak emissions in CH_2_Cl_2_ and toluene suspensions. In addition, the emission maxima of these suspensions varied from 384 nm to 432 nm, showing remarkable blue shift compared to the solid-state fluorescence. The phenomena can most likely be attributed to the different collision interactions rather than crystal structure change [56,57], since that **1** is highly stable in all chosen solvents. Additionally, it is noted that the fluorescence emission intensities are nearly directly proportional to the concentrations of **1** in H_2_O suspensions (Figure S4). 

### 3.5. Fluorescence Sensing of Metal Ions

**Figure 4:**
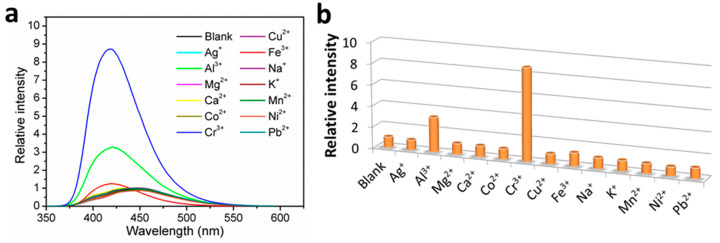
(**a**) Fluorescence emission spectra, and; (**b**) fluorescence relative ratio responses of **1** in H_2_O suspensions containing various metal ions at 1.0 mM.

**Figure 5:**
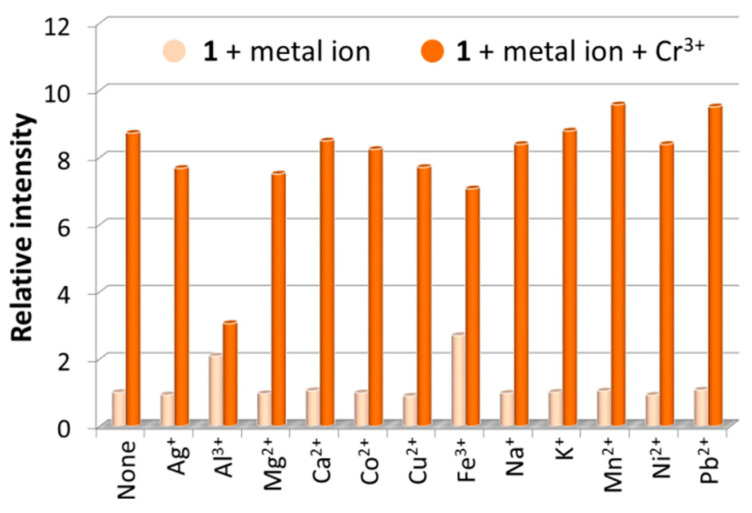
Fluorescence relative ratio responses of **1** in H_2_O suspensions containing various metal ions before and after addition of Cr^3+^ ions with equal concentrations at 1.0 mM.

The fluorescence sensing properties of **1** toward metal ions have been explored, and the fluorescence sensing measurements were carried out in water. Aqueous solutions of nitrate salt of thirteen different metal ions, including Ag^+^, Al^3+^, Mg^2+^, Ca^2+^, Co^2+^, Cr^3+^, Cu^2+^, Fe^3+^, Na^+^, K^+^, Mn^2+^, Ni^2+^, and Pb^2+^, were separately added into the H_2_O suspensions of **1** in a quartz cuvette with the concentration at 1.0 mM. The photoluminescence measurements were obtained at an excitation wavelength of 306 nm before and after addition of metal ions under the same experimental conditions (Figure 4a). Upon addition of the different metal ions, the mono- and divalent metal ions exerted a relatively weak effect (intensity change ≤ 10%) on the emission of **1**, and the Fe^3+^ ion addition led to a weak enhancement effect with ca. 20-nm blue shift. Interestingly, the trivalent metal ions of Cr^3+^ and Al^3+^ resulted in a remarkable fluorescence enhancement by 8.7 and 3.3 times, respectively, along with ca. 20-nm blue shift. The results demonstrate that **1** may be an excellent fluorescence sensor for Cr^3+^ detection with efficient selectivity. To confirm our assumption, interference experiments were carried out to examine the ability of **1** to selectively detect Cr^3+^ ions in the co-existence of interfering metal ions with equal concentrations of 1.0 mM. Experimental results clearly indicated that in sensing Cr^3+^ by **1**, Al^3+^ displayed strong competitive effect while other selected perturbed metal ions showed insignificant interference (Figure 5), suggesting that **1** has good selectivity along with anti-interference ability for Cr^3+^ sensing in water. Briefly stated, **1** is highly selective for Cr^3+^ detection over other perturbed metal ions with the exception of Al^3+^. Further studies on Cr^3+^ detection by varying the concentrations of **1** in H_2_O suspensions showed almost unchanged fluorescence enhancement ratios (Figure S5), suggesting specific Cr^3+^ sensing performances in water.

**Figure 6:**
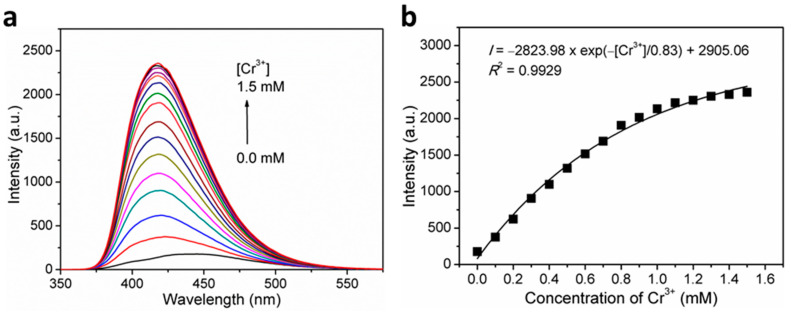
(**a**) Concentration-dependent fluorescence emission spectra of **1** in H_2_O suspensions upon incremental addition of Cr^3+^ ions when excited at *λ*_ex_ = 306 nm, and; (**b**) Plot of fluorescence intensity versus Cr^3+^ ion concentration for **1** in H_2_O suspensions.

To further investigate the sensitivity of **1** toward Cr^3+^ ions, the fluorescence titration experiments were executed. As expected, gradually increasing fluorescence emission intensities were observed at around 420 nm with increasing concentrations of Cr^3+^ ions. As shown in Figure 6b, there exists a nonlinear relationship between the fluorescence intensity and the Cr^3+^ ion concentration, with the formula of *I* = −2823.98 × exp(−[Cr^3+^]/0.83) + 2906.06 (*R*^2^ = 0.9929), suggesting a saturation behavior at high concentrations. On the basis of quantitative titrations (Figure S6), the LOD for Cr^3+^ was determined to be 3.13 μM (corresponding to 162.9 ppb). This proves that **1** can effectively detect Cr^3+^ ions with remarkable sensitivity. 

The possible fluorescence sensing mechanism toward Cr^3+^ was investigated. The XRPD patterns of **1** recovered from Cr^3+^ aqueous solutions showed high consistency with the XRPD patterns of as-synthesized **1** in peak positions (Figure S7), which suggested that the framework of **1** keeps its integrity after Cr^3+^ detection. Thus, the turn-on sensing mechanism can exclude the possibility of framework collapse. However, small but appreciable changes in the relative intensity of the XRPD peaks were observed, so it seems that some changes in the crystal structure occurred. Indeed, X-ray photoelectron spectroscopy (XPS) analysis on **1** indicated the existence of Cr^3+^ cation in the framework of **1** after immersion as the observation of the Cr 2p_3/2_ and Cr 2p_1/2_ peaks at around 577.1 and 586.6 eV, respectively (Figure S8a). This might alter the intensity of the XRPD peaks. Notably, the O 1s peak in the XPS spectra did not shift after Cr^3+^ immersion (Figure S8b), and also the IR spectra did not change significantly (Figure S9). These phenomena imply that the influence of Cr^3+^ is not through bonding or there might be extremely weak interactions only between Cr^3+^ and the framework of **1** instead of the ligand-containing system [47]. Furthermore, the UV−vis absorption spectra of **1** were further checked, which demonstrated that **1** has an absorption band at around 350 nm corresponded to the excitation wavelength applied. Obviously, the absorbance increased remarkably after the addition of Cr^3+^ but exhibited no significant change after the addition of other different metal ions, such as Al^3+^ and Fe^3+^ (Figure S10), which implied that the turn-on effect of **1** toward Cr^3+^ can be properly explained by the absorbance caused enhancement (ACE) mechanism [46,58]. 

### 3.6. Fluorescence Sensing of Anions

**Figure 7:**
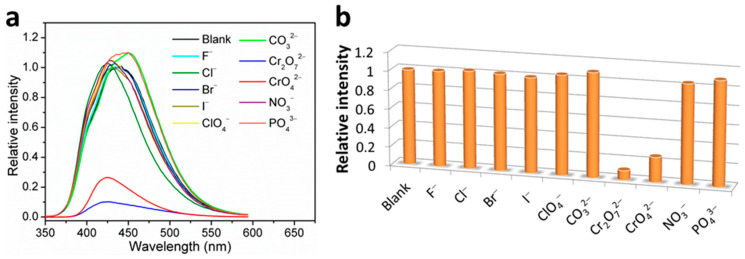
(**a**) Fluorescence emission spectra and (**b**) fluorescence relative ratio responses of **1** in H_2_O suspensions containing various anions at 1.0 mM.

**Figure 8:**
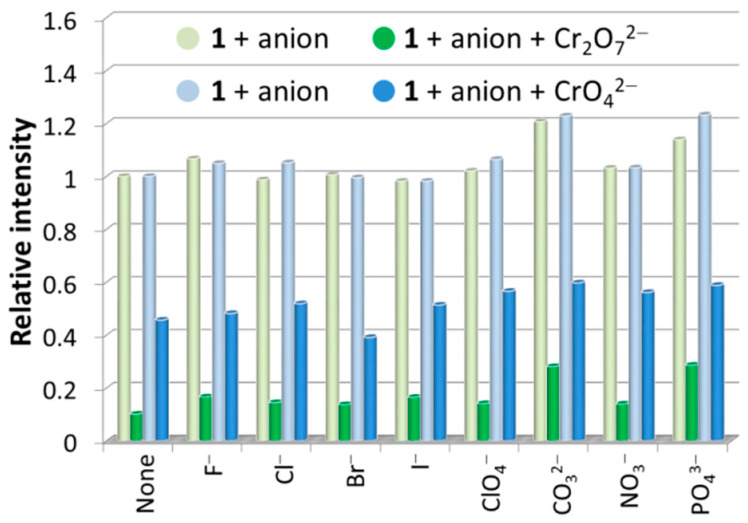
Fluorescence relative ratio responses of **1** in H_2_O suspensions containing various anions before and after addition of Cr_2_O_7_^2−^/CrO_4_^2−^ ions with equal concentrations at 1.0 mM.

The fluorescence sensing properties of **1** toward anions were also explored, and ten different anions, including F^−^, Cl^−^, Br^−^, I^−^, ClO_4_^−^, CO_3_^2−^, Cr_2_O_7_^2−^, CrO_4_^2−^, NO_3_^−^, and PO_4_^3−^, were chosen. Similar to the procedures used for metal ion sensing, the fluorescence sensing measurements were carried out in water; each individual aqueous solution of anion was added to the well-prepared H_2_O suspension of **1**, and the photoluminescence measurements were obtained at an excitation wavelength of 306 nm before and after addition of anion. As can be seen, most of the chosen anions exerted a relatively weak effect (intensity change ≤ 10%) on the emission of **1** (Figure 7). The strongest fluorescence quenching effect was observed in the cases of the two chromium(VI) oxyanions, Cr_2_O_7_^2−^ and CrO_4_^2−^, which showed quenching efficiencies of about 90% and 74%, respectively (quenching efficiency (%) = (*I*_0_ − *I*)/*I*_0_ × 100%, where *I*_0_ and *I* are the maximum fluorescence intensity of **1** before and after addition of analytes). Notably, when different concentrations of **1** in H_2_O suspensions were utilized, the high fluorescence quenching efficiencies are almost retained (Figure S5). Hence, the concentration of **1** in H_2_O suspension has no significant effect on the detection performances toward Cr_2_O_7_^2−^ and CrO_4_^2−^. Furthermore, interference experiments have shown that the quenching efficiencies of **1** toward Cr_2_O_7_^2−^ and CrO_4_^2−^ anions are hardly affected by other competitive anions (Figure 8), confirming the excellent anti-interference ability and thus the high selectivity of **1** as a fluorescence probe for detection of Cr_2_O_7_^2−^ and CrO_4_^2−^ anions in water. 

Since Cr^3+^ enhances fluorescence of **1** in H_2_O suspension and Cr(VI) anions quench it, and both species can coexist in environmental conditions, it is of interest to study the influence of Cr^3+^ detection in the coexistence of Cr(VI) anions and vice versa. Experimental results clearly indicate that Cr(VI) anions strongly interfere with Cr^3+^ detection while Cr^3+^ ions cause no interference on the detection of Cr_2_O_7_^2−^ and CrO_4_^2−^ anions (Figure S11). Again, this confirms that **1** is highly selective for Cr_2_O_7_^2−^/CrO_4_^2−^ detection. 

**Figure 9:**
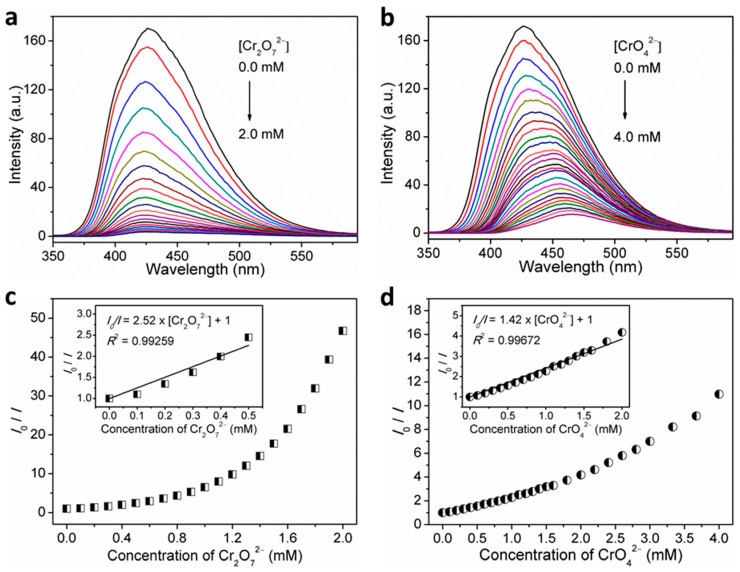
Concentration-dependent fluorescence spectra of **1** in H_2_O suspensions by incremental addition of (**a**) Cr_2_O_7_^2−^, and; (**b**) CrO_4_^2−^ upon excitation at *λ*_ex_ = 306 nm, and Stern–Volmer plot of *I*_0_/*I* versus concentration of; (**c**) Cr_2_O_7_^2−^, and; (**d**) CrO_4_^2−^ for **1** in H_2_O suspensions (inset: linear Stern–Volmer plot).

The detection sensitivity can be determined by quantitative analysis and LOD. Hence, fluorescent titration experiments were performed. As expected, the recorded fluorescence intensities gradually decreased with the gradual increase in the volume concentrations of Cr_2_O_7_^2−^ and CrO_4_^2−^ in the H_2_O suspensions of **1** (Figure 9a,b). Furthermore, the dependence of the fluorescence intensity on Cr_2_O_7_^2−^ or CrO_4_^2−^ ion concentration was investigated, which can be well fitted to *I* = 180.62 × exp(−[Cr_2_O_7_^2−^]/0.56) − 2.86 (*R*^2^ = 0.99717) for Cr_2_O_7_^2−^ and *I* = 154.46 × exp(−[CrO_4_^2−^]/1.06) + 15.61 (*R*^2^ = 0.99789) for Cr_2_O_7_^2−^ (Figure S12). The quantification of fluorescence quenching effect was further examined through the Stern–Volmer equation. As observed, the Stern–Volmer plots for sensing Cr_2_O_7_^2−^ and CrO_4_^2−^ analytes by **1** both exhibited upward curves of *I*_0_/*I* against the analyte concentration over the titration concentrations (Figure 9c,d), implying the cooperation of dynamic and static quenching processes [34,59,60]. On the basis of quantitative titrations, the good linear regression analyses on Stern–Volmer plots gave the *K*_sv_ value of 2.52 × 10^3^ M^−1^ (*R*^2^ = 0.99259) in the range of 0–0.5 mM for sensing Cr_2_O_7_^2−^ and 1.42 × 10^3^ M^−1^ (*R*^2^ = 0.99672) in the range of 0–2.0 mM for sensing CrO_4_^2−^ (inset in Figure 9c,d). The LOD was determined to be 43.36 μM (corresponding to 9.36 ppm) for Cr_2_O_7_^2−^ and 25.57 μM (corresponding to 2.97 ppm) for CrO_4_^2−^ (Figure S13). 

The plausible fluorescence-quenching mechanisms have been investigated. The XRPD patterns of **1** before and after treatment of Cr_2_O_7_^2−^ and CrO_4_^2−^ showed a high degree of similarity (Figure S7), suggesting the maintenance of framework integrity, thus ruling out framework collapse as being the fluorescence quenching mechanism. However, the excitation wavelength to irradiate **1** was greatly overlapped with the absorbance band of Cr_2_O_7_^2−^ and CrO_4_^2−^, implying that the competitive absorption of excitation energy might serve dominant influence on the fluorescence quenching detection of **1** toward Cr_2_O_7_^2−^ and CrO_4_^2−^. Further, energy transfer process might also contribute efforts in quenching the fluorescence of **1** because the fluorescence-emission band of **1** in H_2_O suspension was partially overlapped and the absorbance band of Cr_2_O_7_^2−^ and CrO_4_^2−^ in aqueous solutions (Figure S14). 

## 4. Conclusions

In this research, we have successfully synthesized a 2-fold interpenetrated coordination polymer **1** featuring a 4-connected **cds** network topology with the point symbol of (6^5^·8). Coordination polymer **1** emits fluorescence in both solid-state and suspension-phase of different solvents, making it a potential candidate to be employed in detection of Cr(III) cations via remarkable fluorescence enhancement response due to ACE mechanism, and in sensing of Cr(VI) oxyanions (Cr_2_O_7_^2−^ and CrO_4_^2−^) via fluorescence-quenching effect due to collaboration of absorption competition and energy transfer process, with high sensitivity and selectivity. 

## Figures and Tables

**Table 1:** Crystallographic data for **1**.

	1
Empirical formula	C_31_H_18_BrN_3_O_6_Zn
*M*_w_	673.76
Crystal system	Monoclinic
Space group	*C*2/*c*
*a*, Å	14.254 (2)
*b*, Å	12.566 (2)
*c*, Å	29.985 (5)
*β*, °	102.648 (8)
*V*, Å^3^	5240.2 (15)
*Z*	8
*T*, K	150 (2)
λ, Å	0.71073
*D_cal_*_c_, g cm^−3^	1.708
*F*_000_	2704
*μ*, mm^−1^	2.516
Reflns collected	43704
Unique reflns (*R*_int_)	5360 (0.0751)
Obsd reflns (*I* > 2*σ* (*I*))	4534
Params	379
*R*_1_*^a^*, *wR*_2_*^b^* (*I* > 2*σ* (*I*))	0.0633, 0.1296
*R*_1_*^a^*, *wR*_2_*^b^* (all data)	0.0778, 0.1353
GOF on *F*^2^	1.114
Δρ_max_, Δρ_min_, e Å^−3^	1.312, −0.916

*^a^R*_1_ = $\sum \left|\left|F_{o}\right|-\left|F_{c}\right|\right|/\sum \left|F_{o}\right|$. *^b^* *wR*_2_ = $\{\sum \left[w{\left({F_{o}}^{2}-F_{c}{}^{2}\right)}^{2}\right]/\sum \left[w{\left(F_{o}{}^{2}\right)}^{2}\right]{\}}^{1/2}$.

## Data Availability

All required data is provided within the manuscript.

## Supplementary Materials

The following supporting information can be downloaded at: https://www.mdpi.com/article/10.3390/nano12010158/s1. Figure S1: Plot of the inter-net π–π interactions between two neighboring naphthalimide skeletons in the two independent identical **cds** frameworks in the crystal structure of **1**. Figure S2: TG curve of **1**. Figure S3: Solid-state excitation (solid lines) and emission spectra (solid lines with symbols) of NI-mbpy-34, Br-1,3-H_2_bdc, and **1** at room temperature. Figure S4: Fluorescence emission spectra of **1** in H_2_O suspensions of different concentrations at room temperature upon excitation at 306 nm. Inset: Fluorescence relative ratio responses of **1** in H_2_O suspensions of different concentrations. Figure S5: Effect of the concentration of **1** in H_2_O suspension on the fluorescence intensity responses upon addition of Cr^3+^ ions (enhancement) and Cr(VI) anions (quenching) at 1.0 mM. Figure S6: Linear region of fluorescence intensity for the H_2_O suspensions of complex **1** upon incremental addition of Cr^3+^ ions. The following table lists the relevant parameters of LOD for the H_2_O suspensions of complex **1** toward Cr^3+^ ions. Conditions: *λ_em_* = 414 nm (*λ*_ex_ = 280 nm). Figure S7: XRPD patterns of **1** before and after immersing in Cr^3+^, Cr_2_O_7_^2−^, and CrO_4_^2−^ aqueous solutions for 24 h. Figure S8: (a) XPS high resolution spectra of Cr 2*p* for **1** after sensing Cr^3+^. (b) XPS high resolution spectra of O 1*s* for **1** before and after sensing Cr^3+^. Figure S9: IR spectra of **1** before and after immersing in Cr^3+^ aqueous solution for 24 h. Figure S10: UV-vis spectra of **1** before and after immersing in Cr^3+^, Al^3+^, Fe^3+^ aqueous solutions for 24 h. Figure S11: Fluorescence emission spectra of **1** in H_2_O suspension (1 mg/3 mL) before and after addition of interfering/analyte ions at room temperature upon excitation at 306 nm. Figure S12: Fluorescence intensity traces (*λ*_em_ = 438 nm) for the H_2_O suspensions of **1** upon incremental addition of Cr_2_O_7_^2−^ and CrO_4_^2−^ ions when excited at 306 nm, following the first-order exponential decay. Figure S13: Linear region of fluorescence intensity (*λ*_em_ = 438 nm) for the H_2_O suspensions of **1** upon incremental addition of Cr_2_O_7_^2−^ and CrO_4_^2−^ ions when excited at 306 nm. The following table lists the relevant parameters of LOD for the H_2_O suspensions of **1** toward Cr_2_O_7_^2−^ and CrO_4_^2−^ ions. Figure S14: Spectral overlap between the normalized emission spectra of **1** in H_2_O suspensions and the normalized absorption spectra of Cr_2_O_7_^2−^ and CrO_4_^2−^ in aqueous solutions.
