# Supplementary material for: A Water-Stable 2-Fold Interpenetrating cds Net as a Bifunctional Fluorescence-Responsive Sensor for Selective Detection of Cr(III) and Cr(VI) Ions
Source: Nanomaterials (Basel). 2022 Jan 3;12(1):158. doi: 10.3390/nano12010158 (PMC8746465; doi:10.3390/nano12010158)
Supplement: Supplementary file 1 [file nanomaterials-12-00158-s001.zip › nanomaterials-1491964-supplementary.pdf]

Supporting Information

# A Water-Stable 2-Fold Interpenetrating cds Net as a Bifunctional Fluorescence-Responsive Sensor for Selective Detection of Cr(III) and Cr(VI) Ions

**Meng-Jung Tsai, Kuo-Shun Liao, and Jing-Yun Wu***

Department of Applied Chemistry, National Chi Nan University, Nantou 545, Taiwan. E-mail: jyunwu@ncnu.edu.tw

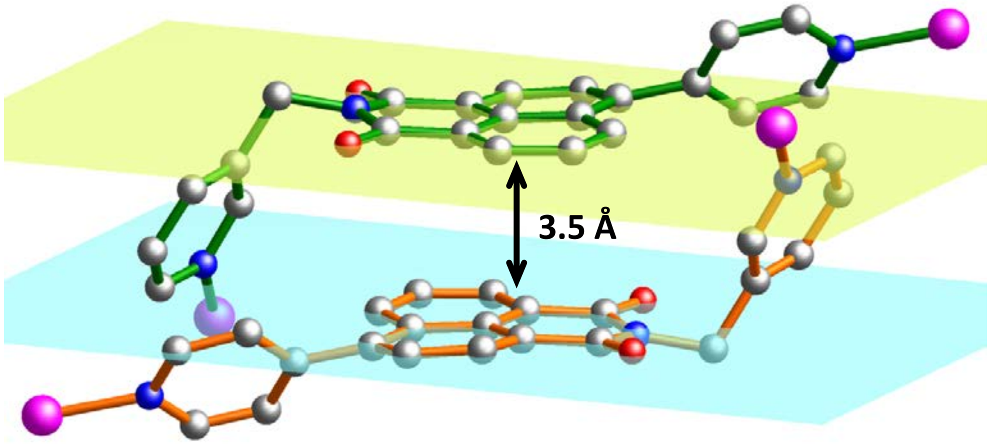


**Figure S1.** Plot of the inter-net π–π interactions between two neighboring naphthalimide skeletons in the two independent identical **cds** frameworks in the crystal structure of **1**.

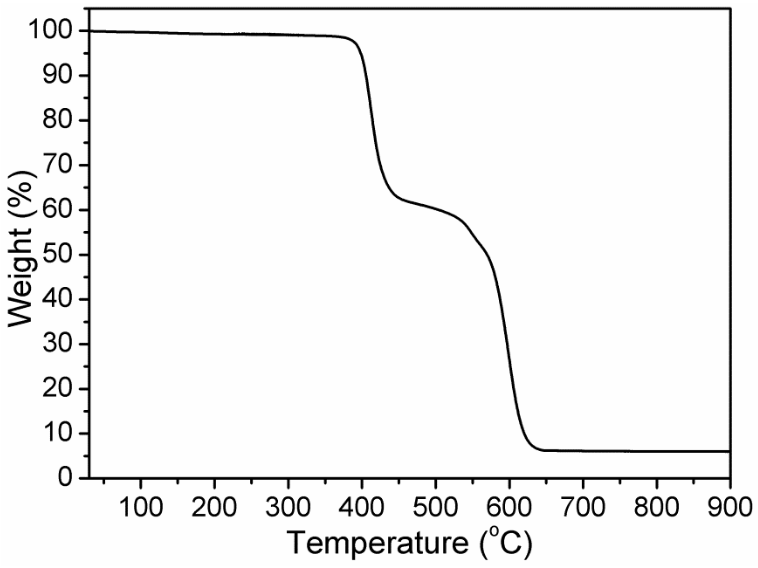


**Figure S2.** TG curve of **1**.

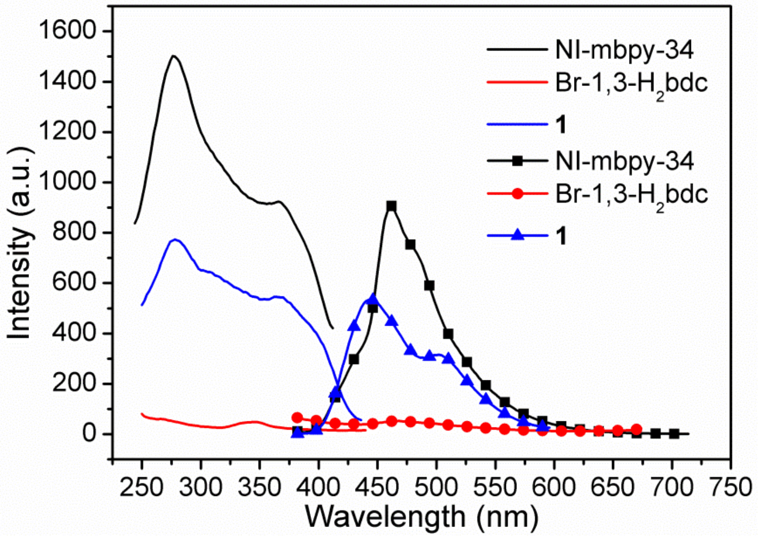


**Figure S3.** Solid-state excitation (solid lines) and emission spectra (solid lines with symbols) of NI-mbpy-34, Br-1,3-$H_2$bdc, and **1** at room temperature.

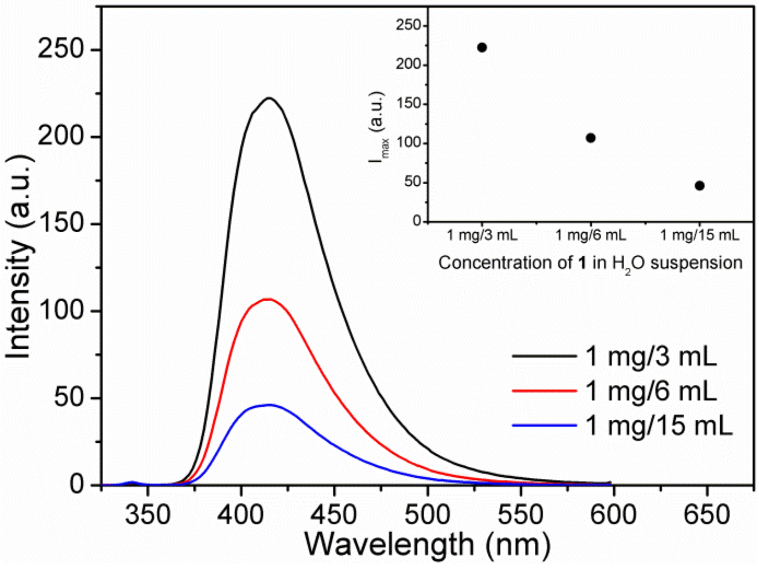


**Figure S4.** Fluorescence emission spectra of **1** in $H_2O$ suspensions of different concentrations at room temperature upon excitation at 306 nm. Inset: Fluorescence relative ratio responses of **1** in $H_2O$ suspensions of different concentrations.

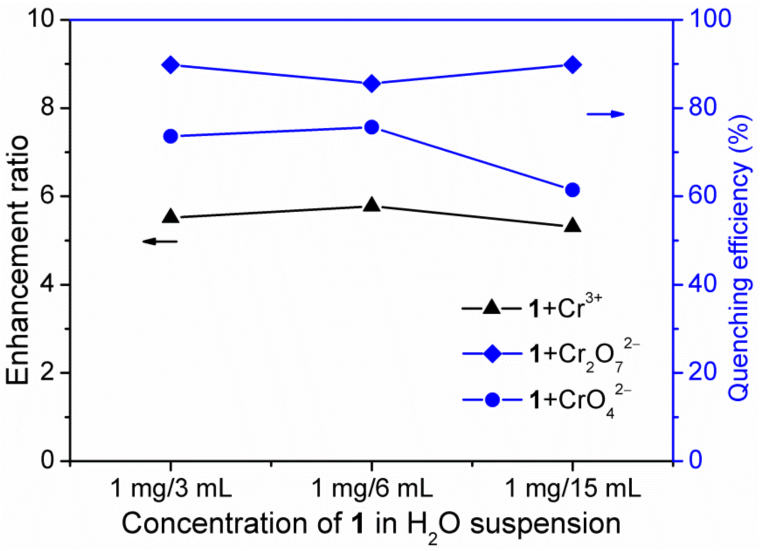


**Figure S5.** Effect of the concentration of **1** in $H_2O$ suspension on the fluorescence intensity responses upon addition of $Cr^{3+}$ ions (enhancement) and Cr(VI) anions (quenching) at 1.0 mM.

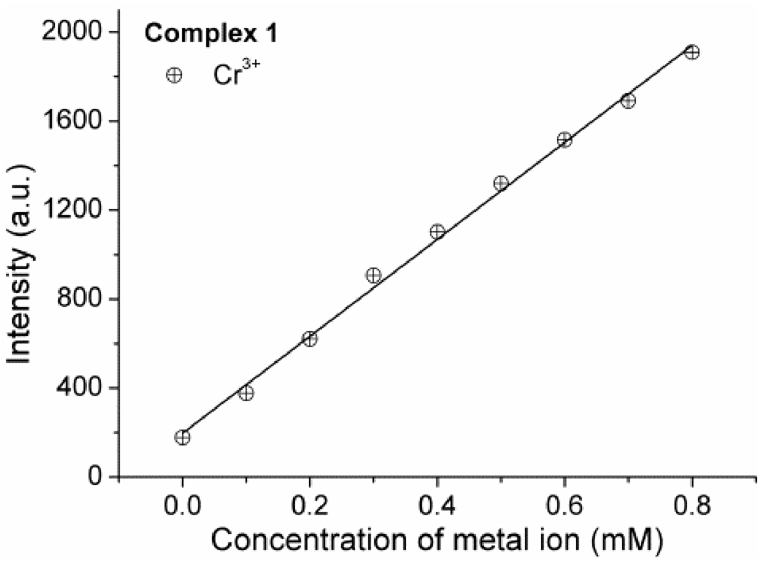


| **Blank readings** | $Cr^{3+}$ |
|---|---|
| #1 | 177.8 |
| #2 | 176.9 |
| #3 | 174.2 |
| #4 | 174.5 |
| #5 | 172.1 |
| **Standard deviation ($\sigma$)** | 2.27 |
| │ **Slope ($k$)** │ | 2178.4 |
| $R^2$ | 0.99631 |
| **LOD ($3\sigma/k$),** μM (ppb) | 3.13 (162.9) |

**Figure S6.** Linear region of fluorescence intensity for the $H_2O$ suspensions of complex **1** upon incremental addition of $Cr^{3+}$ ions. The following table lists the relevant parameters of LOD for the $H_2O$ suspensions of complex **1** toward $Cr^{3+}$ ions. Conditions: $\lambda_{em}$ = 414 nm ($\lambda_{ex}$ = 280 nm).

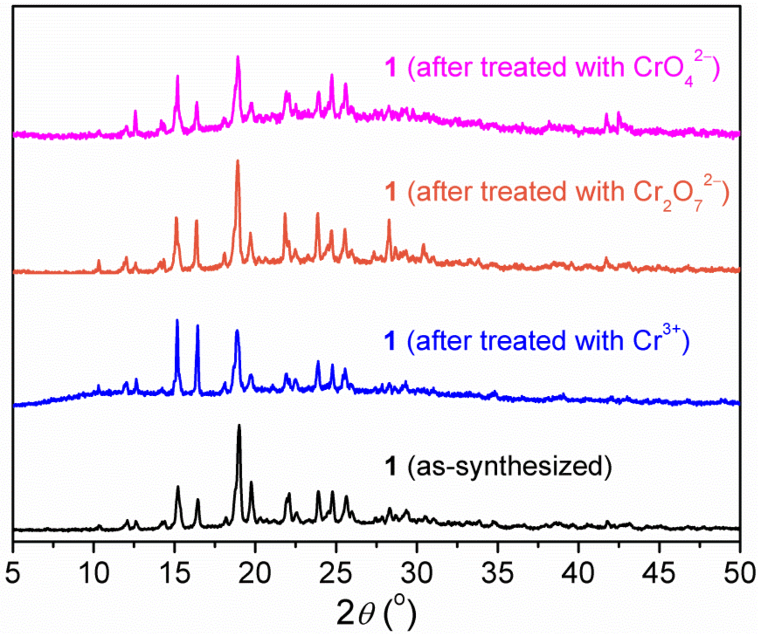


**Figure S7.** XRPD patterns of **1** before and after immersing in $Cr^{3+}$, $Cr_2O_7^{2-}$, and $CrO_4^{2-}$ aqueous solutions for 24 h.

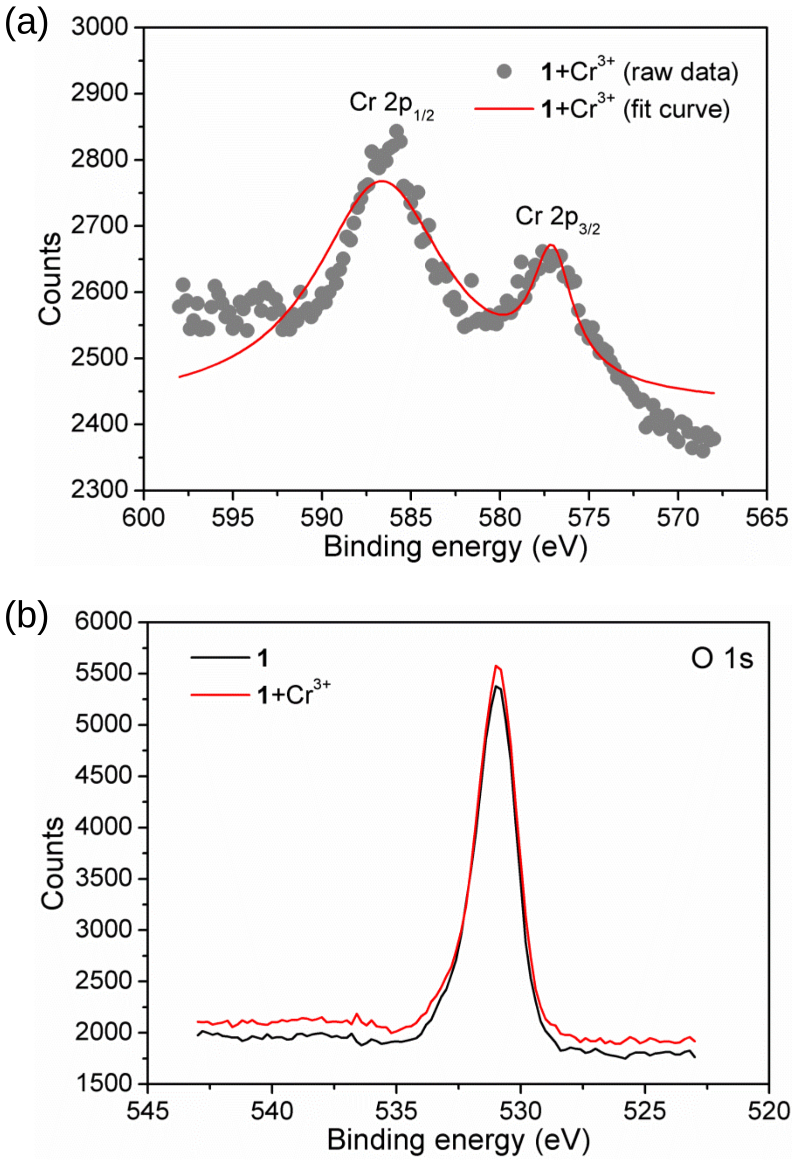


**Figure S8.** (a) XPS high resolution spectra of Cr 2*p* for **1** after sensing $Cr^{3+}$. (b) XPS high resolution spectra of O 1*s* for **1** before and after sensing $Cr^{3+}$.

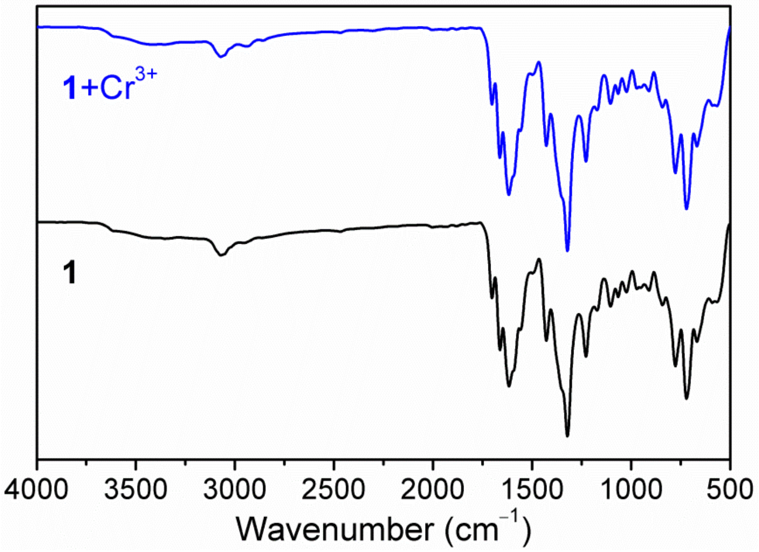


**Figure S9.** IR spectra of **1** before and after immersing in $Cr^{3+}$ aqueous solution for 24 h.

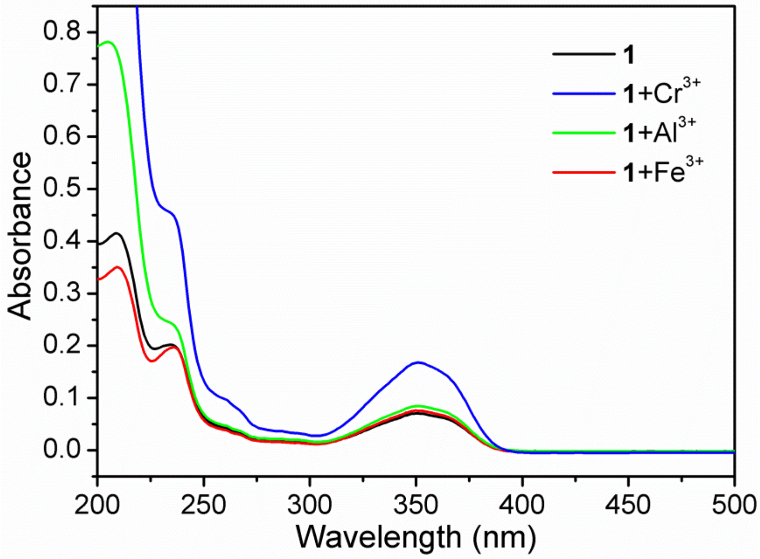


**Figure S10.** UV-vis spectra of **1** before and after immersing in $Cr^{3+}$, $Al^{3+}$, $Fe^{3+}$ aqueous solutions for 24 h.

*Influence of $Cr^{3+}$ detection in the coexistence of Cr(VI) anions and vice versa*

The influence of $Cr^{3+}$ detection in the coexistence of Cr(VI) anions and vice versa using the well-prepared $H_2O$ suspension of **1** (1 mg/3 mL) as sensor was studied. Interference experiments were carried out in two different ways: (1) adding the interfering ions firstly and then adding the analyte ions; (2) adding the mixture of analyte and interfering ions. The concentrations of analyte and interfering ions both are 1.0 mM. Before and after addition of interfering/analyte ions, the photoluminescence spectra were obtained at an excitation wavelength of 306 nm in each step. As shown in Figure S11, experimental results clearly indicate that Cr(VI) anions strongly interfere with $Cr^{3+}$ detection while $Cr^{3+}$ ions cause no interference on the detection of Cr(VI) anions ($Cr_2O_7^{2-}$, $CrO_4^{2-}$).

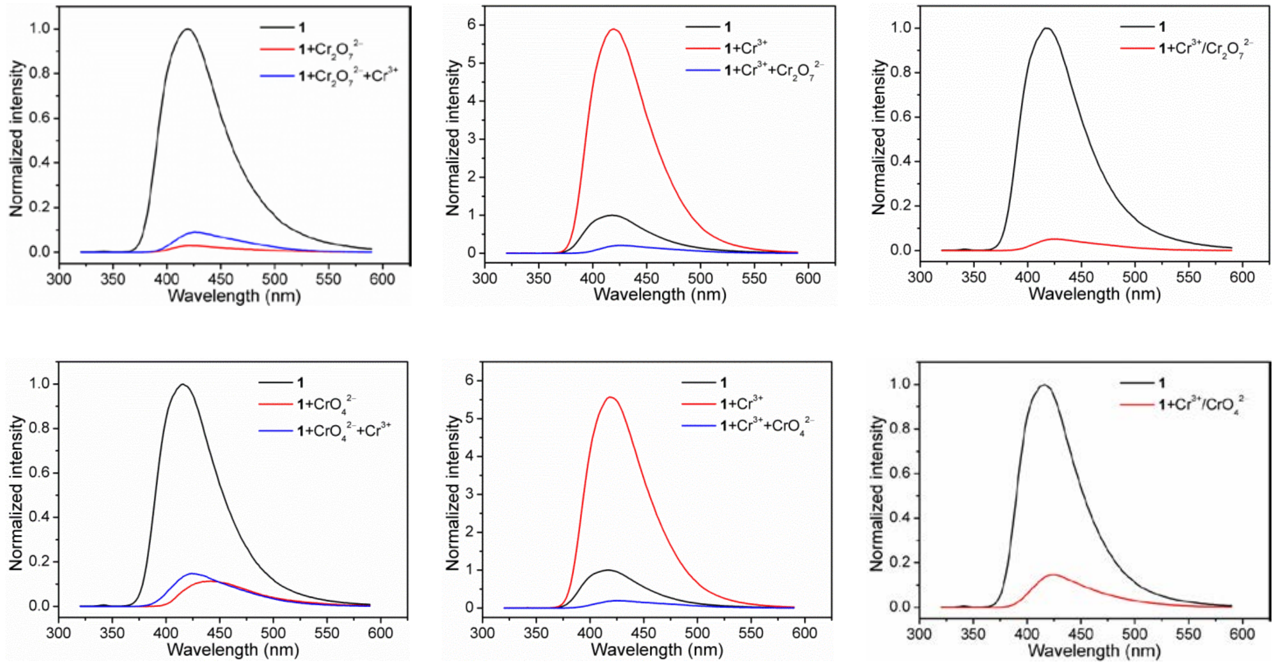


**Figure S11.** Fluorescence emission spectra of **1** in $H_2O$ suspension (1 mg/3 mL) before and after addition of interfering/analyte ions at room temperature upon excitation at 306 nm.

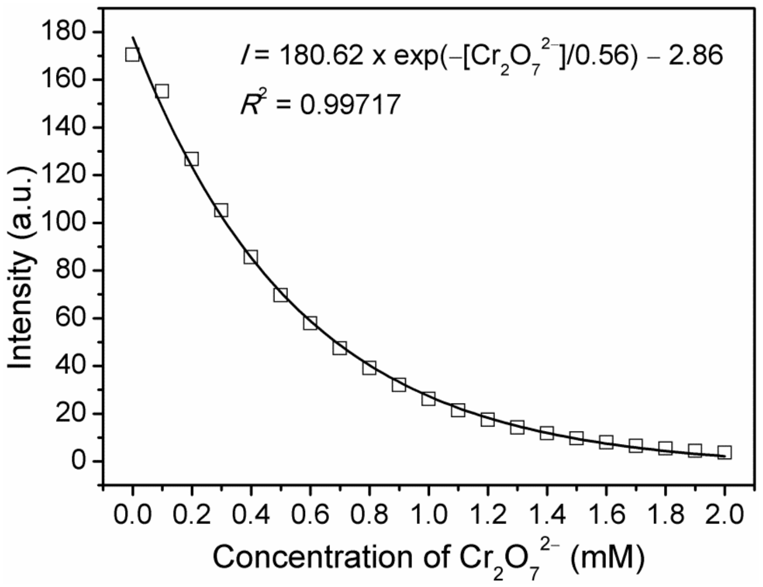


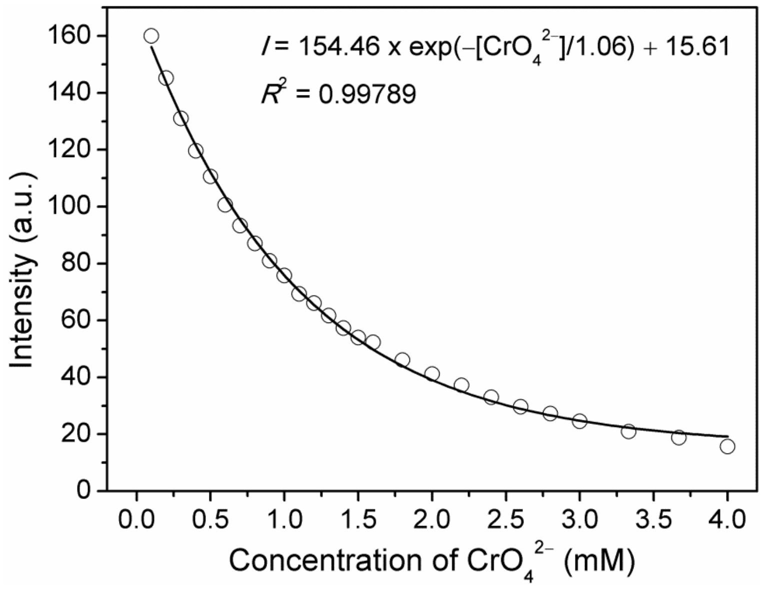


**Figure S12.** Fluorescence intensity traces ($\lambda_{em}$ = 438 nm) for the $H_2O$ suspensions of **1** upon incremental addition of $Cr_2O_7^{2-}$ and $CrO_4^{2-}$ ions when excited at 306 nm, following the first-order exponential decay.

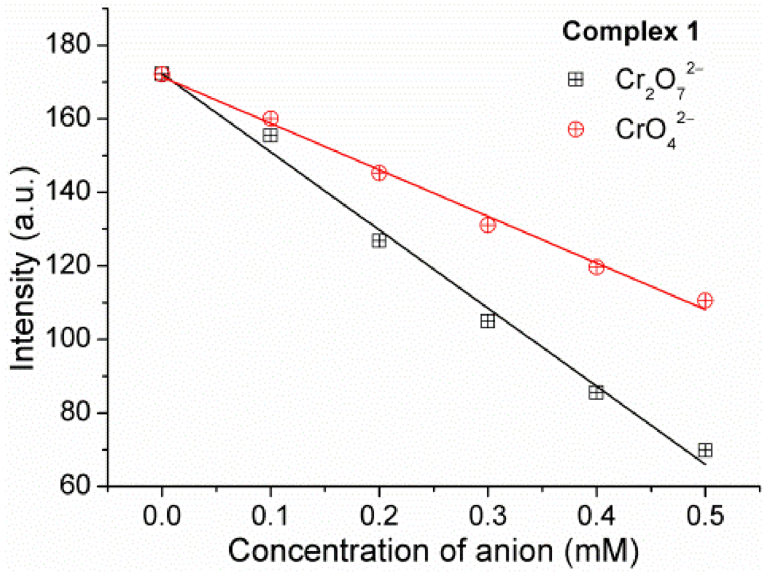


| **Blank readings** | $Cr_2O_7^{2-}$ | $CrO_4^{2-}$ |
|---|---|---|
| #1 | 170.4 | 172.1 |
| #2 | 169.8 | 171.2 |
| #3 | 176.1 | 170.0 |
| #4 | 175.3 | 172.3 |
| #5 | 175.6 | 172.7 |
| **Standard deviation (σ)** | 3.07 | 1.08 |
| $\mid$ **Slope (m)** $\mid$ | 212.4 | 126.5 |
| $R^2$ | 0.99081 | 0.99277 |
| **LOD (3σ/m),** μM (ppm) | 43.36 (9.36) | 25.57 (2.97) |

**Figure S13.** Linear region of fluorescence intensity ($\lambda_{em}$ = 438 nm) for the $H_2O$ suspensions of **1** upon incremental addition of $Cr_2O_7^{2-}$ and $CrO_4^{2-}$ ions when excited at 306 nm. The following table lists the relevant parameters of LOD for the $H_2O$ suspensions of **1** toward $Cr_2O_7^{2-}$ and $CrO_4^{2-}$ ions.

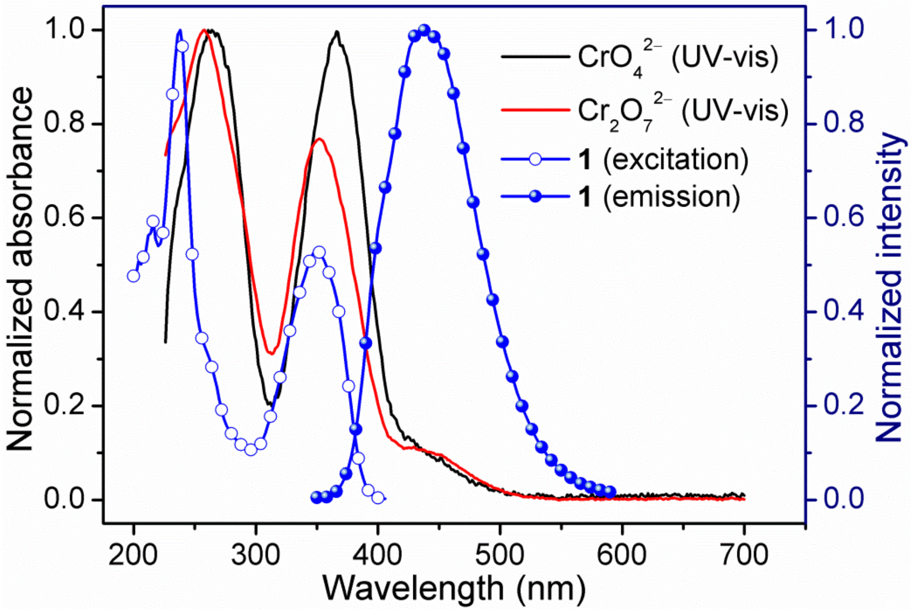


**Figure S14.** Spectral overlap between the normalized emission spectra of **1** in $H_2O$ suspensions and the normalized absorption spectra of $Cr_2O_7^{2-}$ and $CrO_4^{2-}$ in aqueous solutions.
